# Assessing particle and fiber toxicology in the respiratory system: the stereology toolbox

**DOI:** 10.1186/s12989-015-0110-8

**Published:** 2015-10-31

**Authors:** Christina Brandenberger, Matthias Ochs, Christian Mühlfeld

**Affiliations:** Institute of Functional and Applied Anatomy, Hannover Medical School, Carl-Neuberg-Str. 1, 30625 Hannover, Germany; Cluster of Excellence REBIRTH (From Regenerative Biology to Reconstructive Therapy), Hannover, Germany; Biomedical Research in Endstage and Obstructive Lung Disease Hannover (BREATH), Member of the German Center for Lung Research (DZL), Hannover, Germany

## Abstract

The inhalation of airborne particles can lead to pathological changes in the respiratory tract. For this reason, toxicology studies on effects of inhalable particles and fibers often include an assessment of histopathological alterations in the upper respiratory tract, the trachea and/or the lungs. Conventional pathological evaluations are usually performed by scoring histological lesions in order to obtain “quantitative” information and an estimation of the severity of the lesion. This approach not only comprises a potential subjective bias, depending on the examiner’s judgment, but also conveys the risk that mild alterations escape the investigator’s eye. The most accurate way of obtaining unbiased quantitative information about three-dimensional (3D) features of tissues, cells, or organelles from two-dimensional physical or optical sections is by means of stereology, the gold standard of image-based morphometry. Nevertheless, it can be challenging to express histopathological changes by morphometric parameters such as volume, surface, length or number only. In this review we therefore provide an overview on different histopathological lesions in the respiratory tract associated with particle and fiber toxicology and on how to apply stereological methods in order to correctly quantify and interpret histological lesions in the respiratory tract. The article further aims at pointing out common pitfalls in quantitative histopathology and at providing some suggestions on how respiratory toxicology can be improved by stereology. Thus, we hope that this article will stimulate scientists in particle and fiber toxicology research to implement stereological techniques in their studies, thereby promoting an unbiased 3D assessment of pathological lesions associated with particle exposure.

## Introduction

The inhalation of harmful particles can lead to adverse health effects and pathological changes in the respiratory tract. Most often noxious inhaled particles trigger a pulmonary inflammatory response which can initiate the development of sub-chronic or chronic pulmonary diseases including pneumonitis, silicosis, asbestosis, chronic obstructive pulmonary disease (COPD), emphysema, asthma, fibrosis or cancer [1]. The characteristics and severity of particle and fiber-induced pathology depend on exposure time and concentration as well as on particle characteristics such as chemical composition, size, structure and surface composition [2–4]. However, the source and composition of airborne particles is vast - including for example combustion derived particles from traffic and industry, cigarette smoke, silica dust, welding fumes, asbestos, biological particles such as pollen and fungi as well as engineered nanomaterials of various compositions. By dealing with the investigation of multiple physical, chemical and biological parameters at the same time, particle and fiber toxicology becomes a challenging field. Research on respiratory particle toxicology and risk assessment therefore attempts not only to investigate effects of individual particles and sources, but also effects of particle characteristics in general such as shape, size and composition in order to promote predictability of newly generated particles [5]. This is of particular need with the quickly emerging field of nanotechnology and the constant development of new nanoparticles (NP;<100 nm in all three dimensions, ISO/TS 27687:2008) with unknown effects.

Whereas *in vitro* tests are helpful for quick toxicity screening, long term effects in the respiratory tract are usually only assessed by *in vivo* toxicology studies. *In vivo* respiratory toxicology studies also include the histopathological analysis of the lungs to investigate adverse effects of particles. The severity of histopathological lesions indicates the extent of particle toxicity and the type of lesions provide insight into the potential mode of action. Quantitative measurements of histopathological changes in the lungs furthermore enable the calculation of dose–response curves for particle toxicity estimation in risk assessment. Conventional analysis of histopathological lesions usually includes a scoring of the tissue lesion by one or more experienced observers, ideally blinded to the identity of the study group. However, comparative studies have shown that these evaluations are prone to an interpersonal variation with a potential bias [6, 7]. In addition, the degree of alterations has to be large enough to be caught by the investigator’s eye. An accurate way of obtaining quantitative information from histological sections is by the use of stereology. This is an unbiased approach for the quantification of histological structures such as volume, surface area, length and number and has become the gold standard for quantitative microscopy in the respiratory tract [8]. The term stereology is derived from the Greek “stereos” which means spatial and, as a branch of stochastic geometry, the approach is based on solid mathematics [9]. In comparison to other forms of microscopic morphometry – the direct measurement from two dimensional (2D) sections - stereology enables the quantification of the three-dimensional (3D) characteristics of organs, tissues, cells or organelles based on measurements on 2D sections. This is achieved by i) ensuring that each part of the organ has an equal chance of becoming part of the analysis and by ii) applying appropriate test systems/probes to randomly sampled fields of view (see following chapters). The interactions between the structures and the test systems generate counting events that – inserted into the corresponding stereological equations – provide relative values of volume, surface area, length or number of biological structures. By multiplication with the reference volume (e.g. lung volume), total values are attained which are the basis for rigorous statistical testing. This means that in comparison to routine histopathology where only 1–2 tissue sections are analyzed, a larger number of randomly selected lung tissue sections is included in the evaluation (see section for lung stereology below). Thus, this procedure ensures an unbiased and efficient quantitative analysis of the respiratory tract.

Traditionally, lung stereology was developed and applied to assess the structure and function relationships of the lungs [10–12], however, modern experimental morphology and histopathology equally benefit from this unbiased approach [13, 14]. Guidelines to use quantitative histopathology as a biomarker in qualification studies as well as in risk assessment for occupational and environmental health regulations point to the need of unbiased data acquisition, hence stereology for morphometry [15–17]. This review aims at providing an overview of different stereological techniques which can be used to evaluate the severity of respiratory histopathology in particle and fiber toxicology.

### Pathology of pulmonary particle exposure

The pathology of particle and fiber toxicology in the respiratory tract depends on the exposure source, concentration, duration and individual predisposition. Acute responses to particle exposures often include pulmonary inflammation [18]. Various kinds of particle were reported to induce an inflammatory response as for example combustion-derived particles - including diesel exhaust particles [19] and ultrafine particles (< 0.1 μm in aerodynamic diameter) [20], engineered NP such as carbon nanotubes, TiO_2_ or Ag NPs [21–23] as well as silica [24] or asbestos [25]. Chronic particle exposure and long term effects may include the development of chronic obstructive pulmonary disease (COPD) [26–28], allergic airway inflammation [29–34], fibrosis [35–38] or neoplasms [39–41]. In addition, recent studies have also addressed mixed exposures; for example the potential of airborne particles to act as an adjuvant in the development of allergic airway disease or the effect of ozone with combustion derived particles as in the environment [42–46]. Mixture studies include another important aspect of inhalation toxicology which will most likely expand in the future. Table 1 and the following paragraphs give a brief overview on lung pathologies related to particle exposure and how they could be quantified by means of stereology.

**Table 1:** Recommended stereological parameters in different histopathological lesions potentially associated with particle and fiber toxicology

Lung pathology	Histopathology	Stereological parameter
Pulmonary inflammation	Inflammatory cells	Number of inflammatory cells
	Apoptotic cells	Number of apoptotic cells
	Cell proliferation	Number of proliferating cells
	Pulmonary edema (septal, interstitial, alveolar)	Volume of edematous fluid
	Thickening of air-blood barrier	Mean thickness of epithelium, interstitium and endothelium in air-blood barrier (EM)
COPD	Emphysema (I)	Number of alveoli
	Emphysema (II)	Volume of alveolar airspace
	Emphysema (III)	Alveolar surface area
	Septal thickening	Mean septal thickness
	Broncho-epithelial cell hyperplasia	Mean broncho-epithelial thickness
	Mucous cell metaplasia	Mucus per epithelial basement membrane
	Inflammatory cell infiltration	Number of inflammatory cells
Asthma/allergic airway disease	Lymphocyte, Eosinophil, Basophil, Mast cell	Number of inflammatory cells
	Mucous cell metaplasia	Mucus per epithelial basement membrane
	Smooth muscle cell mass	Volume of smooth muscle cells
Fibrosis	Fibroblast hyperplasia	Number of fibroblasts
	Inflammatory cell infiltration	Number of inflammatory cells
	Septal thickening	Mean septal thickness
	Tissue scarring	Volume of non-functional parenchyma
	Collagen deposition	Volume of parenchymal collagen
Cancer	Cell proliferation	Number of proliferative cells
	Tumor cell characteristics	Number of cells positive for tumor marker
	Metastasis (I)	Volume of metastasis
	Metastasis (II)	Number of metastatic nodules

#### Pulmonary inflammation

The extent of pulmonary inflammation and tissue damage can be well addressed via pulmonary histopathology, and different inflammatory parameters can be measured by stereology, including cellular or structural changes. Cellular changes may include the influx of pro-inflammatory cells such as neutrophils and macrophages as well as cellular proliferation or apoptosis. Severe inflammation leads to damage of epithelial or endothelial cells and the exudation of edema fluid to the peri-bronchovascular or alveolar septal interstitium as well as into the alveolar lumen. Stereological parameters which can be used to quantify pulmonary inflammation may include the numbers of inflammatory/proliferating/apoptotic cells, the volume of pulmonary edema or surface area of damaged epithelium/endothelium [13].

#### Asthma/allergic airway disease

The pathology of asthma or allergic airway disease is characterized by airway hyper-responsiveness, reversible airway obstruction, infiltration of eosinophils and CD4^+^ T helper type 2 cells and airway remodeling [47]. Structural changes like infiltration of inflammatory cells and airway remodeling, which may include mucous cell metaplasia, increased smooth muscle mass or sub-epithelial fibrosis, serve as measures of pathological severity of asthma and can be stereologically quantified as cellular numbers, epithelial thickness, the volume of mucus in epithelial cells or the volume of muscle mass and fibrosis [13, 48].

#### COPD/emphysema

The development of COPD is usually triggered by continuous irritation of the lungs resulting in chronic inflammation and airway remodeling, which lead to the development of obstructive bronchiolitis and emphysema [27]. Emphysema is characterized by a distal airspace enlargement and a loss of alveoli and alveolar surface area, which can be assessed by stereological quantification of alveolar number and surface area and/or number-weighted and volume-weighted mean alveolar volume [13, 49, 50]. Obstructive bronchiolitis is assessed in a similar manner to airway obstruction in allergic airway disease.

#### Lung fibrosis

Pulmonary fibrosis is characterized by inflammatory cell infiltration, alveolar epithelial cell injury, fibroblast hyperplasia, collagen deposition and scar formation [51, 52]. Lung fibrosis can be analyzed by quantitative histopathology at light or electron microscopic levels: At light microscopic level for example the volume of nonfunctional parenchyma (collapsed or already remodeled) versus the volume of ventilated parenchyma can be estimated [13], or the volume of parenchymal collagen stained with picrosirius red [53]. At electron microscopic level the thickening of the air-blood barrier can be investigated or the volume of various septal compartments such as collagen, extracellular matrix or fibroblasts [13, 54, 55].

#### Cancer

Assessment of quantitative lung histopathology can help to quantify parameters relevant to tumor development such as the number of proliferative cells, the number or volume of cells staining positive for tumor cell markers or the volume or number of metastatic nodules and carcinomas.

### Quantitative histopathology

**Fig. 1:**
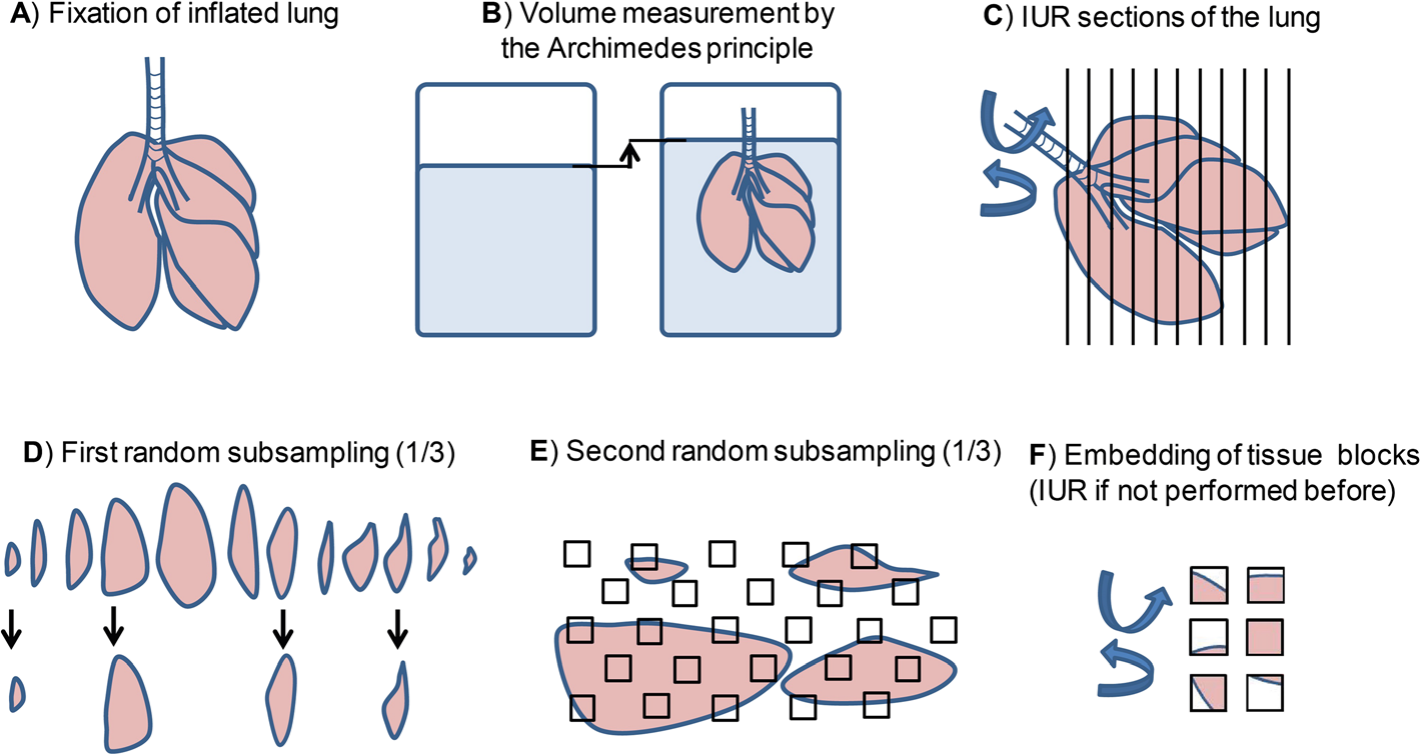
Lung sampling for stereology. **a** Lung fixation under defined inflation pressure. **b** Assessment of the lung volume or reference space by the Archimedes’ principle. **c** Serial sections of the lung – optionally with isotropic uniform random (IUR) orientation with the orientator. **d** Systematic uniform random sampling (SURS) of lung sections: in this example, every 3^rd^ section is included in the sampling. The first section is picked at random - either by throwing a dice or with a random number table. **e** If smaller samples are desired for tissue embedding a further SUR sub-sampling is performed. Again every third tissue piece is selected here and the 1st one chosen randomly. **f** Selected tissue blocks are embedded for LM or TEM – optionally with IUR orientation using the isector

**Fig. 2:**
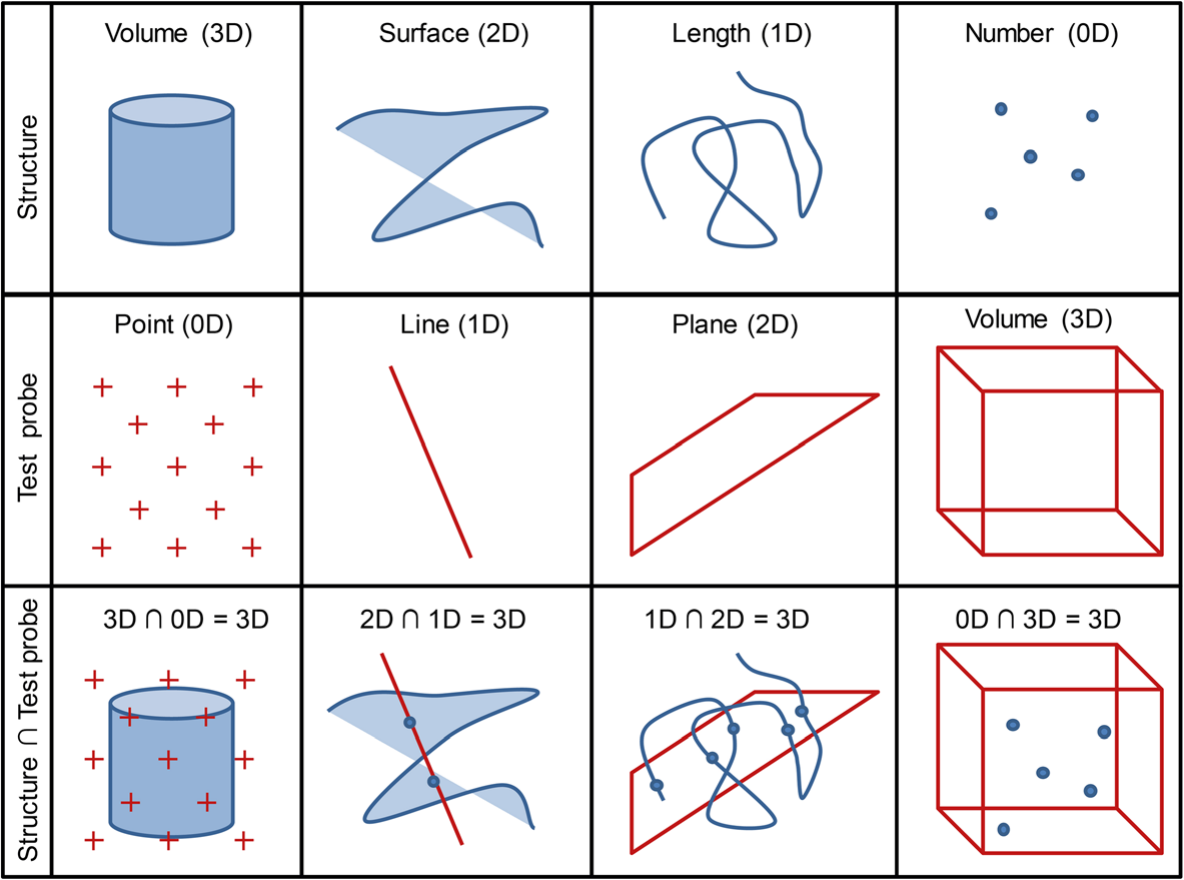
Structures and stereological test probes. The intersection of stereological test probes with the structure of interest provides countable events: test point counts for volumes, test line intersection counts for surfaces, test plane transect counts for length estimation and test volume object counts for number estimation. Note that the sum of the dimension of the test probe and the structure always equals 3. Figure adapted from [96]

The main problem in quantitative histopathology is that tissue sections for microscopic analysis are i) only representing a very small fraction of the whole organ and are ii) more or less 2D. In comparison to other forms of morphometry, which encompasses the direct on-section 2D measurement of structures, design-based stereology takes into consideration the 3D structures of organs, tissues and cells [56]. In order to obtain i) representative information on the whole lung and not only on a single histological section, it is important to give each part of the lung an equal chance of being sampled. Smaller sections from different parts of the lungs are therefore being sampled by an appropriate sampling regime such as systematic uniform random sampling (SURS) as shown in Fig. 1. In order to ii) correct for the loss of one dimension (3D→2D) appropriate test systems are used as shown in Fig. 2. Stereological estimates of parameters such as number, length, surface or volume of the structure of interest are first calculated as densities within their sampling space and multiplied in the end with the volume of the reference space - usually the total lung volume - to obtain total numbers, length, surface area or volume per lung. For these reasons it is very important to be aware that stereological quantification already starts with the collection and sampling of the whole lung.

### Lung sampling for stereology

For stereological analysis, the lungs should be collected and preserved under a controlled inflation pressure (Fig. 1a). Uncontrolled lung inflation pressure during lung fixation can affect the preservation of lung structures and the final lung volume, which are essential for stereological quantification. Sampling and fixation of the lungs is therefore recommended by either inflation fixation via the trachea with fixative application under a defined pressure (recommended pressure 20–25 cm of H_2_O) or by vascular perfusion fixation of an inflated lung under controlled pressure. Generally, inflation fixation is easier to perform and well suited for the estimation of many parenchymal parameters. However, perfusion fixation is better suited for the estimation of vascular and intra-alveolar parameters, including measurements of intra-alveolar edema. In particular, analysis of particle deposition and distribution in the airways and the alveolar lining layer requires the use of fixation by perfusion to ensure that particles can be visualized where they have been deposited. The choice of fixatives such as paraformaldehyde or glutaraldehyde depends on the final method of analysis: If samples are prepared for transmission electron microscopy (TEM), a strong fixative mixture with 1–3% glutaraldehyde is recommended, but for immunohistochemistry, a weaker fixative containing 1–4% paraformaldehyde is suggested. Further information on fixation techniques and embedding methods of choice can be found in [57, 58].

The total lung volume - which represents the reference space in most cases - can be estimated either by the Archimedes’ principle (Fig. 1b) or by the Cavalieri method. For the volume measurement with the Archimedes’ principle, the lungs are immersed in a glass of water until completely covered with water, but not touching the glass (buoyancy). The displaced volume of water equals the volume of the lung and can be estimated by measuring the weight of the displaced water [59]. The Cavalieri method [60] can easily be incorporated during the sampling of the lung (Fig. 1c and d) as described below.

After estimating the lung volume, tissue sections of the lungs are being sampled. As mentioned before, it is important that each part of the lung has an equal probability of being sampled. This guarantees that all data are gathered from a representative sample of the whole lung. Theoretically it is therefore possible to chop the lungs into random pieces of a desired size and independently select a desired number of tissue blocks. However, it has been shown that systematic uniform random sampling (SURS) is more efficient than independent random sampling [61, 62]. Thereby, the lungs are continuously sectioned into slices of approximately the same thickness. The starting point of the first cut is chosen randomly. This ensures the randomness of the sampling. In a next step, a subsampling with a constant sampling interval, as shown in Fig. 1d, is done by including every 2^nd^, 3^rd^, 4^th^ or n^th^ slice into the evaluation and again the first slice is chosen randomly. The choice of subsampling depends on the lung size (species specific) and the microscopic embedding technique (LM or TEM). Hence more subsampling steps are needed for TEM sampling or large lungs, whereas tissue sampling of small lungs (e.g. mice lungs) at LM level might not require any subsampling at all. As a rule of thumb an approximate number of 10 tissue slices is recommended for an unbiased analysis [63]. However, more important than a precise number of tissue sections is that the variation introduced by a number of tissue sections is not greater than among biological individuals. This principle of accuracy versus precision is further discussed in the section on stereological quantification. Random choices can be made simply by throwing a dice, using a random number table or a computer software. The subsampling procedure can be repeated several times, as shown in Fig. 1e and f, until the tissue pieces have the desired size for embedding. In addition to the random selection, the lung samples should also have a random orientation. The lung is an anisotropic organ, meaning that certain lung structures as for example the bronchial tree have a particular spatial orientation. Whereas number and volume estimation are not affected by the anisotropy of the lung, the orientation is critical for the estimation of surface area and length parameters - particularly estimations of the conducting airways and the pulmonary vasculature. Other pulmonary structures such as the parenchyma have no particular spatial orientation and can be regarded as isotropic. In order to avoid any bias due to selective tissue orientation, isotropic uniform random (IUR) orientation is performed at least once during the sampling process in all three axes. This can be done during tissue embedding with the isector [64] or prior to lung sectioning with the orientator [65]. Further details on sampling techniques and systematic uniform random sampling are provided in [66].

### Stereological quantification

Sampling, embedding and sectioning of the lungs for microscopic analysis is followed by the structural measurement. At this point, the three dimensional structure is more or less reduced to a plane, two dimensional section. This means that all structures are reduced by one dimension: a volume is displayed as an area (3D → 2D), an area as a length (2D→1D), a length as a transect (1D→0D) and the zero-dimensional characteristic of a structural number disappears, that means it is not represented in 2D (see Table 2). The principle of stereology takes the loss of dimension into consideration and recovers information on the lost dimension by applying appropriate test probes to the 2D sections. During this process all measurements are expressed as relative values, the so-called densities. Density values are not affected by the loss of one dimension and for example a volume of interest per reference volume in 3D, equals the density of the resulting area of interest per reference area in 2D i.e. mm^2^/mm^2^ = mm^3^/mm^3^ = 1. The same is true for surface area densities or lengths within test fields, i.e. mm/mm^2^ = mm^2^/mm^3^ = mm^−1^ and length densities or transects within test fields, i.e. 1/mm^2^ = mm/mm^3^ = mm^−2^. Upon multiplication with the reference volume (mm^3^), the densities result in volume, surface area or length per lung (Table 2).

**Table 2:** Relationship of stereological test probes and 3D structural quantification of lung pathologies in 2D microscopic images

Parameter in 3D	Parameter 2D in section	Test probe	Counting event	Density	Final measurements
Volume	Area	Test point	Point (P) in test volume	V_V_ = ∑P / total number of test points	V_tot_ = V_V_ x V(ref)
Surface area	Boundary line	Test line	Line intersection (I) with surface area	S_V_ = 2x∑I / total length of test lines	S_tot_ = S_V_ x V(ref)
Length	Transect	Test plane	Transect (Q) with test plane	L_V_ = 2x∑Q / total area of test planes	L_tot_ = L_V_ x V(ref)
Number	-	Disector	Particle event (Q^−^) in test volume	N_V_ = ∑Q^−^ / total disector volume	N_tot_ = N_V_ x V(ref)

As during tissue sampling, it is important that the microscopic fields of view (or images) for the evaluation are chosen randomly. An effective way to ensure this is by applying the principle of SURS. This can easily be done manually by choosing a random starting point outside the sample, followed by step wise left/right and up/down navigation in x and y direction; a so-called meander sampling. It is thereby important that fields of view are strictly selected by chance. Anything like searching for “the greatest lesion” or “best looking area” will create a bias and will jeopardize the scientific value of the study. For light microscopy, there are computer-assisted programs available to perform a random image acquisition.

To ensure an efficient and simple counting procedure, plain geometrical probes are applied such as points, lines or areas. The application of the geometrical probes is displayed in Fig. 2. The general rule for the correct choice of the test probe is that the sum of the dimensions of the structural parameter and the test probe equals three or more, hence suggesting a point grid for volume, test lines for area, a test plane for length and a test volume for number estimation. The latter can be generated by using two thin physical sections or two optical planes from a thick single section, an approach called disector [67] which is explained in more detail in *Examples V and VI*. The geometrical probes are superimposed over the acquired microscopic images. This can also be done digitally with stereology programs like the STEPanizer [68] or manually by generating a transparent foil with the geometrical probe prints which is directly placed on the captured images. The interaction of the geometrical probe with the structure of interest generates a counting event as shown in Fig. 2. The number of counting events is proportional to the “amount” of the structure and the density of the test probe and by knowing the exact dimensions of the geometrical probes, the densities can be calculated as described in Table 2.

Beyond accuracy/unbiasedness, efficiency is also an aim of stereology. Efficiency means that the precision of the data should be balanced between the amount of work (and at which level of the analysis to invest what amount of work) that is needed to gather the data and the precision that is actually needed for the purpose of the study. In order to keep the evaluation efficient it is important to balance the number of tissue blocks, images and counting events in the evaluation. In general it is recommended to generate a total of 100 to 200 counting events per structure of interest from 10 to 15 sections to have a 5–10% coefficient of error [63]. The coefficient of error can be reduced by including a greater number of images in the evaluation or by increasing the density of the test probe, but most of the time it is recommended to include more tissue blocks per lung or lungs per experiment in the evaluation instead. This is reflected by the “Do more less well”- principle (quote by E.R. Weibel, see [69]): it is much more efficient to increase the number of organs or tissue blocks and put less effort into investigating each field of view. The choice of an ideal setup depends on the frequency, distribution and size of the structure of interest. For example, for the quantification of a large, but rare lung lesion, it is recommended to sample more images from several tissue blocks and combine these with a coarse test probe set, rather than using few images with a dense test probe set. The setup of the test system therefore varies from case to case and needs to be designed for the purpose of the current study. It is also worth considering at which level of sampling the largest variation occurs and increasing the sampling at this particular level; e.g. with a high inter-individual variation it is better to increase the number of study subjects or with an irregular lung lesion the number of tissue blocks etc. Details on how to calculate the coefficient of error for a stereological setup can further be found in [14, 63, 70].

**Fig. 3:**
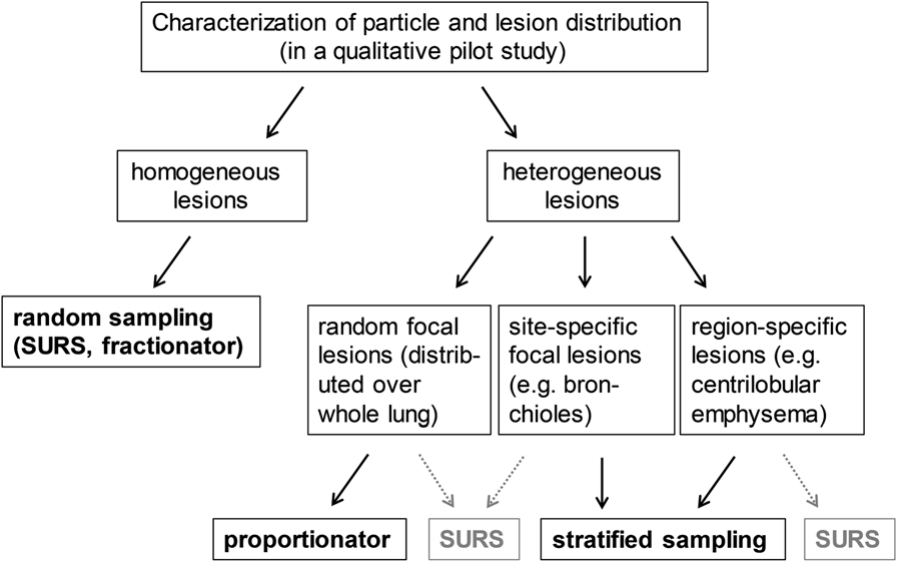
Sampling strategies for homogeneously and heterogeneously distributed lung lesions. Systematic random sampling such as SURS and fractionator sampling are well recommended for homogeneously distributed lung lesions where small sample sizes are sufficient to reach a high precision of the estimate. However, site-specific lesions might not be adequately represented in systematic uniform random sampled tissue or fields of view. Different sampling strategies are therefore recommended for heterogeneous lesions in dependence on lung lesion distribution: Focal lesions which are randomly distributed over the whole lung are best addressed with an initial random tissue sampling followed by the proportionator approach for image acquisition. This enhances the efficiency greatly. If no proportionator is available, a more rigorous image sampling is required to obtain sufficient information as explained in Example IV for the airways. Site-specific lesions as for example in the bronchioles are best approached with stratified sampling in a two-step procedure within randomly sampled histological sections. First, the volume of the compartment of interest is estimated (for example bronchioles) and second, the lesion in the compartment of choice. An example of such a two-step sampling is presented in the Examples II and III for the parenchyma (protocol paragraphs). Note that this approach is still random, though site-specific. SURS and whole lung estimates could still be applied, but are likely to “dilute” the effect; hence subtle pathological changes might be missed. Region-specific lesions such as centrilobular emphysema might be more challenging to assess. If the region-specific lesion can be defined in both control and treated subjects, stratified sampling is recommended. If not, but the lesion is very prominent, SURS is still a valid alternative in combination with pathological description of the region of the lesion. However, certain limitations of the random sampling approach need to be recognized, particularly if the lesions are only very mild and their region not strictly defined

Furthermore, it is worth considering that the deposition and distribution of inhaled particles in the lung depend on particle size and characteristics [71] and do not necessarily follow a random distribution. Non-random particle deposition could lead to site-specific lung lesions [72, 73]. A small pilot study for qualitative characterization of the lung lesions is therefore recommended prior to setting up the stereological study design. With site-specific lung lesions, whole lung SURS, although unbiased, might not be the most efficient approach. Other sampling strategies are therefore recommended for heterogeneous lesions depending on the distribution of the lesion (see Fig. 3). Two methods which are well suited to deal with site-specific lung lesions in stereology without jeopardizing an unbiased histopathological quantification are stratified sampling [66] or the proportionator [74, 75]. The principle of stratified sampling includes a step of subdivision into different “strata” or compartments of the lung. Each compartment is then sampled by SURS and evaluated according to its content. Thereby the sampling efficiency in a specific compartment can be enhanced and the evaluation related to a specific compartment of interest. The proportionator is another elegant approach which combines conventional stereology with automatic image analysis and can be applied for quantifying site-specific lesions with an inhomogeneous distribution within the lung. The proportionator operates at the level of image sampling for stereological quantification by automatically selecting a structure of interest - for example a specific cell type stained with IHC - in proportion to its occurrence. The quantification efficiency is increased by a proportional oversampling of the structure of interest, with known probability, thus an unbiased quantification is still maintained by correcting for the known oversampling. In comparison to conventional stereology, the proportionator requires digitalization of histological slides and suitable software for automatic image analysis. Further literature on the theory and application of the proportionator can be found in [74–76].

### Protocols

**Fig. 4:**
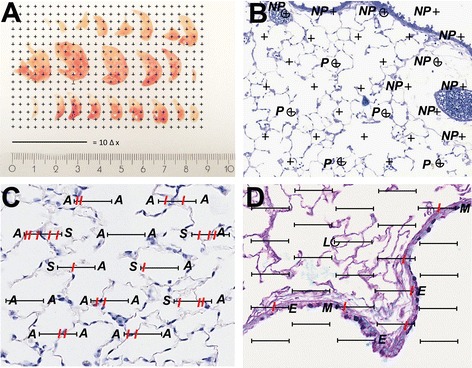
Example of volume and surface area estimation with stereological probes. **a** Lung volume estimation by Cavalieri method: a point grid with a known area per point is superimposed over mouse lung sections. The number of points multiplied by the area per point and the slice thickness will result in the total lung volume. **b** Volume estimation of parenchymal (P) and non-parenchymal (NP) lung volume by point counts. Note that a four-fold coarse point grid was included for the counting of parenchymal points. **c** Volume estimation of alveolar septa (S) and airspace (A) by point counts and estimation of alveolar surface area with line probe intersections (I). **d** Quantification of mucus per length of basement membrane on AB/PAS mucus positive section (purple). The volume of mucus (M) and epithelium (E + M) is estimated with point counts and the surface area of the basement membrane with the line probe intersections (I). A fifty-fold coarse point grid was included for the counting of lung tissue points (L)

**Fig. 5:**
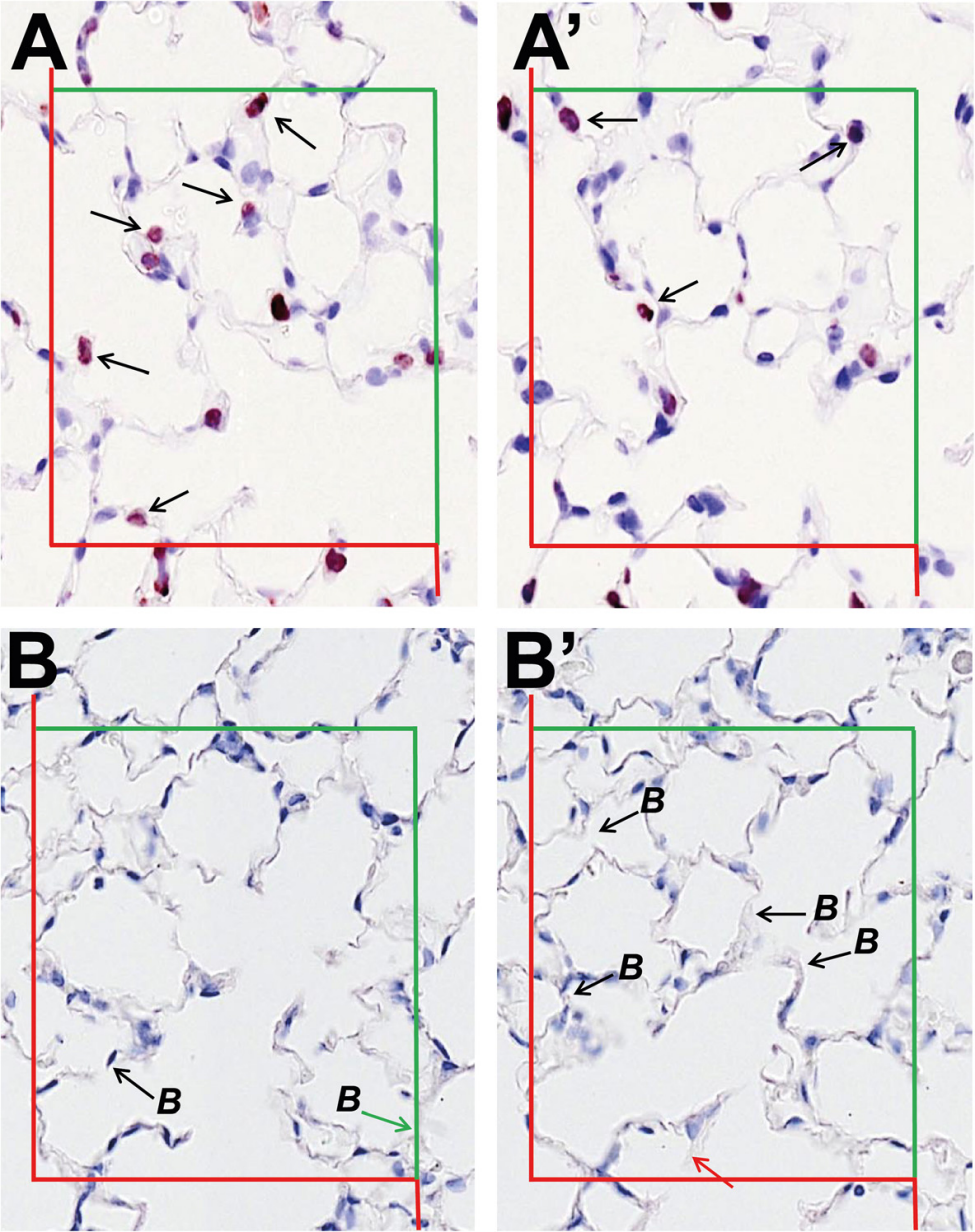
Number estimation with disector. **a** Cell number estimation with the disector. Proliferating cells (BrdU positive) are stained with immunohistochemistry in brown. All cells within the counting frame which are present on the reference section (**a**) but not on the look-up section (**a**’) are counted (arrow) and vice versa. **b** Alveolar number estimation with the disector. Bridges (B) are counted in the reference section (**b**) and look-up section (**b’**). Note that any bridges in touch with the red exclusion line are not included (red arrow) in the evaluation and those in touch with the green inclusion line are (green arrow)

Depending on the histopathological structure of interest, various stereological strategies can be applied to quantify particle induced lesions. Some examples could be a) volume of the whole lung, the parenchyma, fibrotic tissue or specific cells, b) surface area of alveolar epithelium and capillary endothelium, c) mean thickness of alveolar septa or tracheobronchial epithelium and d) number of inflammatory cells or alveoli. Selected examples (Figs. 4 and 5) with calculations (Table 3) are demonstrated in the following section. Further detailed examples and calculations can be found in [13].

**Table 3:** Examples of stereological calculations

Estimation (formula)	Counts (example)^a^	Results (example)^a^
Cavalieri (Fig. 4a)	$$ \begin{array}{ll}\sum P\hfill & =125\hfill \\ {}a/p\hfill & ={\left(3.5\ mm\right)}^2\hfill \\ {}d=\hfill & 3\ mm\hfill \end{array} $$	$$ \begin{array}{ll}V(lung)\hfill & =4594\ m{m}^3\hfill \end{array} $$
*V*(*lung*) = ∑*P x a*/*p x d*		
Parenchymal Volume (Fig. 4b)	$$ \begin{array}{ll}\sum P(par)\hfill & =218\times 4=872\hfill \\ {}\sum P(nonpar)\hfill & =107\hfill \\ {}\sum P(lung)\hfill & =\sum \left(P(par)+P(nonpar)\right)\hfill \\ {}\hfill & =979\hfill \end{array} $$	$$ \begin{array}{ll}V\left(par, lung\right)\hfill & =4092\ m{m}^3\hfill \\ {}V\left( nonpar, lung\right)\hfill & =502\ m{m}^3\hfill \end{array} $$
$$ \begin{array}{ll}V\left(par, lung\right)\hfill & =\sum P(par)/\sum P(lung)\times V(lung)\hfill \\ {}V\left( nonpar, lung\right)\hfill & =\sum P(nonpar)/\sum P(lung)\times V(lung)\hfill \end{array} $$		
Alveolar volume, surface area and septal thickness (Fig. 4c)	$$ \begin{array}{ll}\sum I\hfill & =394\hfill \\ {}\sum P(sept)\hfill & =94\hfill \\ {}\sum P(alv)\hfill & =333\hfill \\ {}\sum P(par)\hfill & =94+333=427\hfill \\ {}l/p\hfill & =35\ \mu m\hfill \end{array} $$	$$ \begin{array}{ll}{V}_V\left( sept/par\right)\hfill & =0.22\hfill \\ {}{V}_V\left(alv/par\right)\hfill & =0.78\hfill \\ {}{S}_V\left( sept/par\right)\hfill & =527.3\ c{m}^{-1}\hfill \end{array} $$
$$ \begin{array}{ll}{V}_V\left( sept/par\right)\hfill & =\sum P(sept)/\sum P(par)\hfill \\ {}{V}_V\left(alv/par\right)\hfill & =\sum P(alv)/\sum P(par)\hfill \\ {}{S}_V\left( sept/par\right)\hfill & =\left(2\times \sum I\right)/\left(\sum P(par)\times l/p\right)\hfill \\ {}S\left( sept, lung\right)\hfill & ={S}_V\left( sept/par\right)\times V(par)\hfill \\ {}V\left(alv, lung\right)\hfill & ={V}_V\left(alv/par\right)\times V(par)\hfill \\ {}\tau (sept)\hfill & =2\times {V}_V\left( sept/par\right)/{S}_V\left( sept/par\right)\hfill \end{array} $$		$$ \begin{array}{ll}V\left(alv, lung\right)\hfill & =3191.8\ m{m}^3\hfill \\ {}S\left( sept, lung\right)\hfill & =2158\ c{m}^2\hfill \\ {}\tau (sept)\hfill & =8.3\ \mu m\hfill \end{array} $$
Epithelial mucous cell metaplasia (Fig. 4d)	$$ \begin{array}{ll}\sum I\hfill & =294\hfill \\ {}\sum P(muc)\hfill & =89\hfill \\ {}\sum P(epi)=\hfill & 245\hfill \\ {}\sum P(lung)\hfill & =396\times 50=19800\hfill \\ {}l/p\hfill & =35\ \mu m\hfill \end{array} $$	$$ \begin{array}{ll}{V}_V\left(muc/ lung\right)\hfill & =0.0045\hfill \\ {}{V}_V\left(epi/ lung\right)\hfill & =0.012\hfill \\ {}{S}_V\left(bm/ lung\right)\hfill & =0.85\ m{m}^{-1}\hfill \end{array} $$
$$ \begin{array}{ll}{V}_V\left(muc/ lung\right)\hfill & =\sum P(muc)/\sum P(lung)\hfill \\ {}{S}_V\left(bm/ lung\right)\hfill & =\left(2\times \sum I\right)/\left(\sum P(lung)\times l/p\right)\hfill \\ {}V/S\left(muc/bm\right)\hfill & =\left(\sum P(muc)\times l/p\right)/\left(2\times \sum I\right)\hfill \\ {}\tau (epi)\hfill & ={V}_V\left(epi/ lung\right)/{S}_V\left(bm/ lung\right)\hfill \end{array} $$		$$ \begin{array}{ll}V/S\left(muc/bm\right)\hfill & =5.3\ \mu {m}^3/\mu {m}^2\hfill \\ {}\tau (epi)\hfill & =14.1\ \mu m\hfill \end{array} $$
Cell numbers (Fig. 5a)	$$ \begin{array}{ll}\sum {Q}^{-}\hfill & =107\ \left( both\ ways\right)\hfill \\ {}n\hfill & =53\hfill \\ {}A\hfill & =200\times 250\ \mu {m}^2\hfill \\ {}h\hfill & =5\ \mu m\hfill \end{array} $$	$$ \begin{array}{ll}{N}_V\left( cell/ lung\right)\hfill & =4038\ m{m}^{-3}\hfill \\ {}N\left( cell, lung\right)\hfill & =18.5\times {10}^6\hfill \end{array} $$
$$ \begin{array}{ll}{N}_V\left( cell/ lung\right)\hfill & =\sum {Q}^{-}/\left(2\times n\times A\times h\right)\hfill \\ {}N\left( cell, lung\right)\hfill & =NV\left( cell/ lung\right)\times V(lung)\hfill \end{array} $$		
Alveolar number (Fig. 5b) $$ \begin{array}{ll}N\left(alv, lung\right)\hfill & =\left(\sum B\right./\left.\left(2\times n\times A\times h\right)\right)\times V(lung)\hfill \end{array} $$	$$ \begin{array}{ll}\sum B\hfill & =112\ \left( both\ ways\right)\hfill \\ {}n\hfill & =90\hfill \\ {}A\hfill & =200\times 250\ \mu {m}^2\hfill \\ {}h\hfill & =5\ \mu m\hfill \end{array} $$	$$ \begin{array}{ll}{N}_V\left(alv/ lung\right)\hfill & =2489\ m{m}^{-3}\hfill \\ {}N\left(alv, lung\right)\hfill & =11.4\times {10}^6\hfill \end{array} $$

^a^The values presented in the following examples are related to Figs. 4 and 5. All density calculations are multiplied with the lung volume as estimated with the Cavalieri method in Fig. 4a to obtain estimates of volume, surface area and number of structure of interest. Note that the calculations are based on examples from rat lungs, but results might dissent from expected values and no shrinking corrections were applied in the formulas

#### Volume estimation

##### Example I: Lung volume by the Cavalieri method

An example for the use of test points to estimate a volume is the Cavalieri method for the estimation of the reference volume: After sectioning of the lung into slices of roughly the same thickness, all lung slices are placed laterally with the same orientation. A point grid with a defined area represented by each point (a/p) is randomly placed on the lung slices and the points hitting the cut surface of the lung sections are counted (Fig. 4a). The total number of point counts on the lung tissue (P) is multiplied with the area per point and the section thickness (d). The multiplication results in the total lung volume (V(lung)) as shown in eq. 1:

1 $$ V(lung) = {\displaystyle \sum }P\times a/p\times d $$

##### Example II: Parenchymal volume

The lung parenchyma represents the gas exchanging region of the lung, including alveolar airspace and the capillary containing septa. Especially chronic exposure to harmful particles and fibers can lead to severe changes in the lung parenchyma including fibrosis or emphysema. The value of parenchymal volume itself does not provide sufficient information to characterize and quantify parenchymal lesions, but it may be used as a reasonable first impression of the extent of the lesions, particularly when it is combined with other estimates such as alveolar surface area or alveolar number. The lung parenchymal volume is best quantified at a lower magnification such as 5x or 10x. After SURS image acquisition, a point grid is superimposed over the images and all points on the parenchyma (P(par)) and non-parenchyma (P(nonpar)) are counted. The volume of the parenchyma is then calculated by dividing the number of points hitting the parenchyma by the number of points hitting the reference volume (P(lung)) and multiplied by the total lung volume as shown in eq. 2. Since the ratio of parenchyma versus non-parenchyma is around 9:1 it is recommended to use a coarse and a fine counting grid to attain 100–200 counting events each and to keep the workload reasonable. A double test system combining a coarse and a fine point grid as shown in Fig. 4b, can be used for this purpose. Hereby all coarse points on the parenchyma and all fine points on the non-parenchyma will be counted at the same time. However, it is important to keep the ratio of coarse to fine points in mind and multiply the coarse points with the inverse ratio before including them in eq. 2. The same principle of volume estimation can be applied for the quantification of other structures as, for example the parenchymal collagen in fibrotic lungs or the alveolar airspace in lung emphysema.

2 $$ V\left(par, lung\right)=\kern0.5em {\displaystyle \sum }P(par)\ /{\displaystyle \sum }P(lung) \times V(lung) $$

#### Surface area and volume estimation

##### Example III: Alveolar volume and surface area

The total alveolar surface area gives an estimate of the functional gas exchange area and is measured with test lines. At the same time as the alveolar surface area, the alveolar airway volume, the septal volume and the mean septal thickness can be estimated. The latter can directly be derived from septal volume and surface area [77, 78] and could be a measure of alveolar septal thickening as occurring in interstitial lung diseases. A SURS image sampling at a 20x magnification is recommended for this evaluation. A test line system, as displayed in Fig. 4c, is superimposed and the intersections (I) of the test line segments with the alveolar septa are counted as well as the number of line endpoints - which serve in this case as a point grid - on the alveolar lumen (P(alv)) and on the septa (P(sept)). The total alveolar airspace volume is calculated by dividing the number of points hitting alveolar airspace by the total number of points hitting the parenchymal reference area (∑(P(alv) + P(sept)) and multiplying the result with the parenchymal lung volume as described above. The surface density of alveolar septa (S_V_(sept/par)) is calculated by relating the total number of intersections of the test lines with alveolar septa to the total number of points hitting the reference volume and to the length of test line associated with each point of the test system (l/p) as shown in eq. 3. If only one of the line end points is used for point counting, l/p equals the length of an individual test line segment. The total septal surface area (S(sept,lung)) is calculated by multiplying S_V_(sept/par) with V(par) as in eq. 4.

3 $$ {S}_V\left( sept/par\right)=\left(2\times {\displaystyle \sum }I\right)/\left({\displaystyle \sum }P(par)\times l/p\right) $$

4 $$ S\left( sept, lung\right)={S}_V\left( sept/par\right)\times V(par) $$

The mean septal thickness (τ(sept)) can be calculated from the septal volume density (V_V_(sept/par)) and the septal surface density (S_V_(sept/par)) according to eq. 5.

5 $$ \tau (sept)=2\times {V}_V\left( sept/par\right)/{S}_V\left( sept/par\right) $$

##### Example IV: Epithelial mucous cell metaplasia

Various studies have shown that combustion-derived particles and nanoparticles can act as adjuvants in the development of allergic airway disease [30, 33, 42]. A histopathological feature of allergic airway disease in different species is epithelial mucous cell metaplasia [79] and the quantification of mucous cell metaplasia can serve as a measure in the assessment of the adjuvant potential of particles in allergy development [33, 42]. The mucosubstance in broncho-epithelial cells can be quantified per lung as well as per basement membrane of broncho-epithelial cells, where the basement membrane serves as an internal reference measure [80, 81]. A SURS image acquisition is recommended at 20x magnification. Since the conducting airways are part of the non-parenchymal fraction of the lung, which is much smaller than the parenchymal fraction, a more rigorous image sampling of 150 to 200 images might be required or alternatively the application of the proportionator [74]. For the analysis, a line test probe is superimposed over the images (Fig. 4d). All intersections (I) with the bronchial epithelial basement membrane are counted as well as all line endpoints hitting the epithelium (P(epi)), the intracellular mucosubstance (P(muc)) and on the lung tissue (P(lung)). A coarse sub-sampling as shown in Fig. 4d is recommended for counting the lung points, to maintain the evaluation efficient. If doing so, the counted lung points need to be multiplied by the inverse sub-sampling fraction in order to obtain the total lung points (ΣP(lung)). The mucus density (V_V_(muc/lung)) is calculated by dividing all points hitting the mucus by the reference points (ΣP(lung)) as shown in eq. 6. The surface density of the epithelial basement membrane (S_V_(bm/lung)) is estimated as shown in eq. 7 and the volume of mucus per unit surface area of basement membrane V/S(muc/bm) is calculated as shown in eq. 8.

6 $$ {V}_V\left(muc/ lung\right)={\displaystyle \sum }P(muc)/{\displaystyle \sum }P(lung) $$

7 $$ {S}_V\left(bm/ lung\right)=\left(2\times {\displaystyle \sum }I\right)/\left({\displaystyle \sum }P(lung)\times l/p\right) $$

8 $$ V/S\left(muc/bm\right)=\kern0.5em \left({\displaystyle \sum }P(muc)\times l/p\right)/\left(2\times {\displaystyle \sum }I\right) $$

The mean thickness of airway epithelium (τ(epi)) – a measure of epithelial hyperplasia and hypertrophy - can furthermore be calculated by dividing the volume density of epithelial cells by the surface density of the basement membrane as shown in eq. 9.

9 $$ \tau (epi)={V}_V\left(epi/ lung\right)/{S}_V\left(bm/ lung\right) = \left({\displaystyle \sum }P(epi)\times l/p\right)/\left(2\times {\displaystyle \sum }I\right) $$

#### Number estimation with the disector

Number estimation can be used to quantify discrete objects or “particles” such as cells or alveoli. As mentioned before, each structure is reduced by one dimension in a 2D microscopic section and a discrete object is not represented/countable any more in a thin single section. In order to compensate for the loss of dimension, a disector pair is used, consisting of two (usually consecutive) sections with a known distance height (h) [67, 82]. These sections can either be generated from two thin physical sections (physical disector) or optical sections (from z-stack images; optical disector). The latter might be obtained by focusing and imaging through one thick light microscopic section [83] or by selecting optical sections from a 3D tomography [84]. Particularly laser scanning microscopy (LSM) is very well suited for the application of the optical disector, since image z-stacks naturally generate multiple disector pairs [83, 85]. Thereby, a volume will be recreated – which is the volume between the surface of the first and the surface of the second section. As discrete objects with an easy and regular surface topology (such as cells or nuclei) have only one beginning or end in vertical height, the number of tops or bottoms contained in the volume between the two sections is proportional to the number of objects within a unit of the reference volume. The criterion to count an object in a disector is that it is present in one section (reference section) but not in the other section (look-up section). The distance between the two sections of a disector – the disector height - should be roughly one third of the average object size and not larger than the smallest particles, otherwise the object may be lost between the two sections. The volume reconstruction for object counting requires the knowledge of the distance (h) between the two sections and an area wherein the objects are counted. The counting area is specified by an unbiased counting frame [86] of a known area (A) and only cells within the frame are part of the evaluation. To avoid any over- or under-sampling in the test field area, the unbiased counting frame consists of two inclusion and two exclusion lines and their extensions (green and red in Fig. 4a and b, respectively). Any cells touching the exclusion line are omitted from the evaluation whereas the cells touching the inclusion line are counted. Further theoretical and practical details on the disector can be found in [13, 63, 67].

##### Example V: BrdU positive cell counts

An application of the disector is the estimation of the number of particular cells of interest in the lung. Especially the number of inflammatory cells could be useful for analyzing the effects of particle and fiber exposure. Another potentially relevant example is the quantification of proliferative cells as a result of injury and repair which can be visualized by Bromodesoxyuridine (BrdU) application and immunohistochemistry [82]. Figure 5a shows a disector pair of lung parenchyma stained for BrdU positive proliferative cells. The cells present in the reference section (Fig. 5a), but not in the look-up section (Fig. 5a’), are marked with arrows. Only the BrdU positive cells are counted which are not present in the look-up section (for example 5 counts in Fig. 5a and 3 counts in Fig. 5a’). The test field is specified by an unbiased counting frame [86] of a known area (A) and only cells within the frame are part of the evaluation. The red line of the counting frame marks the forbidden line and any cell in touch with the red line is excluded from the evaluation whereas the cells on the green line are included. The numerical cell density (N_V_(cell/lung)) is then calculated by dividing the number of counting events (Q^−^) by the number of evaluated test fields (n), the counting frame area (A) and the distance height between the disector pairs (h) as shown in eq. 10. The number of test fields (n) on the lung tissue can be estimated for example by counting all edges of the counting frame hitting the lung tissue and dividing the obtained counts by four.

10 $$ {N}_V\left( cell/ lung\right)={\displaystyle \sum }{Q}^{-}/\left(n\times A\times h\right) $$

If the cellular profiles are counted both ways (as shown in Fig. 5a) the resulting density needs to be further divided by two. The total number of BrdU positive cells in the lung is obtained by multiplying the numerical cell density by the total lung volume.

##### Example VI: Alveolar number

The loss of alveolar number is a measure for the development of emphysema as for example in cigarette smoke induced COPD [87]. The estimation of the numerical density and the total number of alveoli in the lung can be achieved with the disector method. The alveolar number is estimated by counting the alveolar openings – which represents a single event per alveolus - at the level of the free septal edges, where they form a two-dimensional network [88]. The alveolar network and number can further be estimated with the Euler number [88, 89]. The Euler number (χ) is obtained by subtracting “Bridges” (B) from “Islands” (I) and “Holes” (H) [90]. A bridge is a structure which connects two alveolar septa and closes an open alveolus as shown in Fig. 5b. Islands and holes can be disregarded in the alveolar network. Note that bridges which touch the exclusion line or its extension are not included in the evaluation (Fig. 5b’ red arrow) but those touching the green inclusion line are part of the evaluation (Fig. 5b green arrow). The alveolar numerical density (N_V_(alv/lung)) is calculated by dividing the number of bridges (B) by the total test volume as described in *Example V* and shown in eq. 11. Again, if counts are performed both ways, the density needs to be divided by two. The total alveolar number per lung is obtained by multiplying the alveolar density number with the total lung volume (V(lung)). More information and details on the Euler connectivity in lung stereology can be found in [88, 89].

11 $$ {N}_V\left(alv/ lung\right) = {\displaystyle \sum }B/\left(n\times A\times h\right) $$

### Particle deposition and uptake in lung cells

In addition to quantitative histopathology, stereology can also be applied to quantify particle distribution and uptake in lung cells or cell cultures [91–93]. To estimate the deposition and uptake of particles in the lung, the particle number or volume distribution within the lung, at organ, tissue, cellular or organelle level can be quantified by relating their occurrence within a specific compartment of interest to the volume of this compartment. This stereological approach is called relative deposition index [94]. However, particles must be unambiguously identifiable in the tissue or cells by the microscopic techniques of choice [95]. The microscopic technique of choice is dependent on particle characteristics and may include polarized LM, LSM, TEM or energy filtered TEM (reviewed in [58]). Some particle types may not be suited for microscopic quantification at all due to poor visualization and limited resolution capacities.

### Challenges and pitfalls

It is important to note that unbiased stereological data can only result from unbiased data acquisition. Most pitfalls resulting in biased data acquisition occur during sample preparation, e.g. lung fixation, reference space measurement or tissue embedding and sectioning [96]. Some pitfalls which require particular attention are listed below:

1. Controlled lung inflation: Uncontrolled inflation during the fixation of the lungs results in distortion of dimensions and hence biased quantification. To avoid such artifacts, the inflation pressure of the lung needs to be monitored during fixation - either by controlling the pressure of fixative application during inflation fixation or by monitoring the air and perfusion pressure in the lungs during perfusion fixation. The lungs might be immersed in fixative for a couple of hours or days till further processing to ensure complete fixation of the tissue. It is recommended to keep the preservation time in fixative constant within an experiment to avoid structural changes over time.
2. Measuring the reference space: A missing reference space or lung volume estimation furthermore eliminates the possibility of total lung structure measurements and only allows relative measurements. Interpretations based on ratio densities, without knowledge of changes in the reference space (the so-called reference trap) are frequent and can be misleading [97]. It is therefore most important to never forget to measure the reference space.
3. Unbiased tissue sampling: An unbiased random sub-sampling of the lungs is crucial to ensure that every part of the lung has an equal chance of being sampled. Furthermore, it is important to keep in mind that surface and length estimations also require spatial random orientation. Random orientation in space can be acquired for example by the isector [64] or the orientator [65].
4. Tissue deformation: All quantitative parameters are distorted by tissue deformation. Certain fixatives and embedding media such as formalin fixation and paraffin embedding are prone to result in tissue shrinkage and deformation. There are different solutions to circumvent this problem such as using different fixatives and embedding media or monitoring the extent of shrinkage. Fixatives and resins as used in TEM embedding present less tissue shrinkage [57]. However, certain stains and section preparations might not work on glutaraldehyde fixed and resin embedded samples. Alternatively it is recommended to monitor tissue shrinkage. This can be done by embedding a tissue piece of known dimensions and tracking the extent of shrinkage over the embedding and sectioning process. The percent of tissue shrinkage then needs to be corrected in the application of density calculations in all dimensions. Further information on the issue of tissue shrinking in stereological measurements can be found in [98].
5. Biopsies: Particularly in human lung pathology often only small biopsy samples are available. Lung biopsy samples require a special handling since they do not meet most criteria for lung stereology: biopsy sites are usually non-random, the tissue might be collapsed, tissue fixation and preparation might be compromised due to specific requirements for diagnostic analysis and the reference space is restricted to the biopsy and not the whole organ [8]. All these constraints are a limitation for conducting unbiased stereology. However, some principles of stereological measurements can still be applied for relative structural quantification. Measurements can be performed by applying a suitable internal reference space and stereological test probes can be applied to quantify the structures of interest. Further details or examples on how to handle biopsies can be found in [99–101].

## Conclusions

The severity and mode of action of noxious particles and fibers as well as other inhalative toxicants can be assessed with pulmonary histopathology. For this reason pulmonary histopathology serves as an important instrument in toxicity studies and for risk assessment. With the use of stereology, histopathological lesions can be quantified in an efficient, yet unbiased manner, allowing the creation of dose response curves and estimating effect levels based on lesions and pathologies. In combination with computer programs designed for stereology the quantification can be further facilitated. The current review aimed to provide an overview on different particle and fiber associated lung pathologies and how stereology can be implemented in their quantitative evaluation. The examples given serve as an illustration on how to approach stereology in respiratory toxicology, and common pitfalls in quantitative histopathology are discussed. We hope that this article will stimulate scientists in particle and fiber research to implement stereological techniques in their studies in order to improve the quality of morphometric quantification.

## Abbreviations

2D: Two dimensional
3D: Three dimensional
A: Area
a/p: Area per point
alv: Alveolar
BrdU: Bromodesoxyuridine
COPD: Chronic obstructive pulmonary disease
d: Section thickness
h: Disector height
I: Intersection
L: Length
LM: Light microscope
LSM: Laser scanning microscope
l/p: Length per point
L_V_: Length density
N: Number
nonpar: Non-parenchymal
NP: Nanoparticle
N_V_: Number density
P: Point
par: Parenchyma
PM10: Particulate matter (<10 μm)
Q: Count in 2D
Q^−^: Count in 3D(disector)
S: Surface
sept: Septal
S_V_: Surface density
SURS: Systematic uniform random sampling
TEM: Transmission electron microscope
τ: Thickness
V: Volume
V_V_: Volume density
χ: Euler number
